# Supplementary material for: Identification of potential saliva and tear biomarkers in primary Sjögren’s syndrome, utilising the extraction of extracellular vesicles and proteomics analysis
Source: Arthritis Res Ther. 2017 Jan 25;19:14. doi: 10.1186/s13075-017-1228-x (PMC5264463; doi:10.1186/s13075-017-1228-x)
Supplement: Additional file 1: Table S1. — Upregulated proteins in whole saliva from patients with pSS. Table S2. Upregulated proteins in EVs isolated from whole saliva from patients with pSS. Table S3. Upregulated proteins in tear fluid from patients with pSS (DOC 348 kb) [file 13075_2017_1228_MOESM1_ESM.doc]

**Table S**1. Upregulated proteins in whole saliva of pSS patients

| **Upregulated proteins in pSS patients** | **Gene name** | **T-test**  **(p-value)** | **Spectral count controls** | **Spectral count**  **pSS** |
| --- | --- | --- | --- | --- |
| Neutrophil gelatinase-associated lipocalin | LCN2 | 0.00019 | 3 | 37 |
| Ig gamma-1 chain C region | IGHG1 | 0.00052 | 70 | 132 |
| Fibrinogen alpha chain | FGA | 0.0012 | 1 | 20 |
| Ig lambda-2 chain C regions | IGLC2 | 0.0014 | 111 | 94 |
| Zymogen granule protein 16 homolog B | ZG16B | 0.0016 | 15 | 37 |
| Galectin-3-binding protein | LGALS3BP | 0.0022 | 8 | 33 |
| Ig kappa chain V-I region Ni | KV121 | 0.0031 | 2 | 15 |
| Fibrinogen gamma chain | FGG | 0.0034 | 9 | 48 |
| Matrix metalloproteinase-9 | MMP9 | 0.0044 | 16 | 55 |
| Clusterin | CLU | 0.0051 | 17 | 30 |
| Transketolase | TKT | 0.0059 | 37 | 56 |
| Plastin-2 | LCP1 | 0.0072 | 20 | 68 |
| Beta-2-microglobulin | B2M | 0.0072 | 6 | 20 |
| Calmodulin | CALM1 | 0.0073 | 1 | 17 |
| Ig kappa chain V-III region HIC | KV313 | 0.0084 | 10 | 21 |
| Calmodulin-like protein 5 | CALML5 | 0.011 | 0 | 13 |
| Granulins | GRN | 0.012 | 0 | 13 |
| Rho GDP-dissociation inhibitor 1 | ARHGDIA | 0.016 | 0 | 7 |
| Annexin A1 | ANXA1 | 0.018 | 16 | 30 |
| Ig kappa chain C region | IGKC | 0.021 | 174 | 199 |
| Calmodulin-like protein 3 | CALML3 | 0.021 | 7 | 23 |
| Alpha-enolase | ENO1 | 0.022 | 69 | 94 |
| L-lactate dehydrogenase B chain | LDHB | 0.022 | 0 | 7 |
| Hemopexin | HPX | 0.023 | 40 | 60 |
| Ig kappa chain V-II region TEW | KV204 | 0.025 | 32 | 34 |
| Epididymal secretory protein E1 | NPC2 | 0.029 | 0 | 11 |
| Adenylyl cyclase-associated protein 1 | CAP1 | 0.029 | 12 | 21 |
| Ig gamma-2 chain C region | IGHG2 | 0.029 | 36 | 82 |
| Glyceraldehyde-3-phosphate dehydrogenase | GAPDH | 0.032 | 42 | 69 |
| Gelsolin | GSN | 0.04 | 49 | 82 |
| Heat shock 70 kDa protein 1A | HSPA1A | 0.04 | 33 | 60 |
| WAP four-disulfide core domain protein 2 | WFDC2 | 0.04 | 5 | 19 |
| Olfactomedin-4 | OLFM4 | 0.04 | 0 | 13 |
| Glutathione S-transferase P | GSTP1 | 0.04 | 33 | 55 |
| Complement C3 | C3 | 0.044 | 66 | 151 |
| Metalloproteinase inhibitor 1 | TIMP1 | 0.046 | 9 | 17 |
| Thioredoxin | TXN | 0.045 | 3 | 16 |
| Brain acid soluble protein 1 | BASP1 | 0.049 | 5 | 11 |

**Table S**2. Upregulated proteins in EVs isolated from whole saliva of pSS patients

| **Upregulated proteins in pSS patients** | **Gene name** | **T-test**  **(p-value)** | **Spectra**  **count**  **controls** | **Spectral**  **count**  **pSS** |
| --- | --- | --- | --- | --- |
| HLA class I histocompatibility antigen, B-15 alpha chain | HLA | 0.0012 | 15 | 35 |
| Protein S100-A9 | S100A9 | 0.0016 | 63 | 121 |
| HLA class I histocompatibility antigen, B-7 alpha chain | HLA-B | 0.0023 | 4 | 12 |
| Catalase | CAT | 0.0026 | 5 | 18 |
| Lymphocyte antigen 6D | LY6D | 0.0028 | 5 | 12 |
| TYRO protein tyrosine kinase-binding protein | TYROBP | 0.0037 | 0 | 6 |
| Neutrophil defensin 1 | DEFA1 | 0.0073 | 18 | 22 |
| Transforming protein RhoA | RHOA | 0.009 | 16 | 31 |
| Guanine nucleotide-binding protein subunit alpha-13 | GNA13 | 0.0092 | 1 | 8 |
| Antileukoproteinase | SLPI | 0.0094 | 21 | 27 |
| Annexin A6 | ANXA6 | 0.096 | 78 | 138 |
| Tyrosine-protein phosphatase non-receptor type substrate 1 | SIRPA | 0.01 | 0 | 9 |
| Putative neutrophil cytosol factor 1B | NCF1B | 0.01 | 4 | 18 |
| WD repeat-containing protein 1 | WDR1 | 0.011 | 1 | 11 |
| Mucin-5AC | MUC5AC | 0.011 | 0 | 43 |
| Actin-related protein 2/3 complex subunit 1B | ARPC1B | 0.011 | 24 | 36 |
| Lymphocyte-specific protein 1 | LSP1 | 0.012 | 0 | 9 |
| Ig lambda chain V-III region LOI | LV302 | 0.012 | 8 | 11 |
| CD59 glycoprotein | CD59 | 0.012 | 46 | 51 |
| High affinity immunoglobulin epsilon receptor subunit gamma | FCER1G | 0.012 | 2 | 8 |
| Protein XRP2 | RP2 | 0.012 | 0 | 5 |
| Neutrophil gelatinase-associated lipocalin | LCN2 | 0.015 | 3 | 17 |
| Voltage-gated hydrogen channel 1 | HVCN1 | 0.015 | 0 | 6 |
| Ig kappa chain V-III region HAH | KV312 | 0.015 | 2 | 8 |
| Glucose-6-phosphate isomerase | GPI | 0.016 | 2 | 14 |
| Receptor-type tyrosine-protein phosphatase eta | PTPRJ | 0.016 | 6 | 21 |
| HLA class I histocompatibility antigen, A-24 alpha chain | HLA-A | 0.017 | 0 | 14 |
| Synaptosomal-associated protein 23 | SNAP23 | 0.017 | 0 | 6 |
| Ig heavy chain V-I region HG3 | HV102 | 0.017 | 3 | 8 |
| Brain acid soluble protein 1 | BASP1 | 0.018 | 16 | 46 |
| Adipocyte plasma membrane-associated protein | APMAP | 0.02 | 0 | 26 |
| Guanine nucleotide-binding protein G(k) subunit alpha | GNAI3 | 0.021 | 3 | 18 |
| Ras-related protein Rab-5C | RAB5C | 0.021 | 0 | 7 |
| Intercellular adhesion molecule 3 | ICAM3 | 0.024 | 3 | 7 |
| Carbonic anhydrase 4 | CA4 | 0.025 | 4 | 17 |
| Mucin-2 | MUC2 | 0.025 | 0 | 27 |
| Protein HIDE1 | HIDE1 | 0.026 | 0 | 5 |
| Receptor-type tyrosine-protein phosphatase C | PTPRC | 0.028 | 46 | 86 |
| Sodium- and chloride-dependent neutral and basic amino acid transporter B(0+) | SLC6A14 | 0.029 | 0 | 5 |
| Alpha-actinin-1 | ACTN1 | 0.029 | 15 | 35 |
| Serine/threonine-protein phosphatase PP1-alpha catalytic subunit | PPP1CA | 0.029 | 0 | 5 |
| CD9 antigen | CD9 | 0.03 | 10 | 13 |
| Cornifin-A | SPRR1A | 0.031 | 16 | 34 |
| Calmodulin | CALM1 | 0.031 | 6 | 21 |
| Fermitin family homolog 3 | FERMT3 | 0.032 | 19 | 36 |
| Basigin | BSG | 0.032 | 1 | 8 |
| 2',3'-cyclic-nucleotide 3'-phosphodiesterase | CNP | 0.034 | 0 | 6 |
| Ubiquitin-60S ribosomal protein L40 | UBA52 | 0.034 | 28 | 30 |
| Talin-1 | TLN1 | 0.035 | 10 | 33 |
| F-actin-capping protein subunit beta | CAPZB | 0.038 | 35 | 53 |
| Coronin-1A | CORO1A | 0.039 | 23 | 33 |
| Destrin | DSTN | 0.039 | 0 | 7 |
| Na(+)/H(+) exchange regulatory cofactor NHE-RF2 | SLC9A3R2 | 0.04 | 0 | 5 |
| Ig kappa chain V-III region NG9 (Fragment) | KV303 | 0.041 | 5 | 8 |
| Sialic acid-binding Ig-like lectin 5 | SIGLEC5 | 0.041 | 1 | 8 |
| Olfactomedin-4 | OLFM4 | 0.044 | 18 | 77 |
| Guanine nucleotide-binding protein G(I)/G(S)/G(T) subunit beta-2 | GNB2 | 0.045 | 44 | 76 |
| Beta-arrestin-2 | ARRB2 | 0.046 | 0 | 6 |
| Peptidoglycan recognition protein 1 | PGLYRP1 | 0.046 | 9 | 20 |
| Neutrophil cytosol factor 2 | NCF2 | 0.047 | 7 | 12 |

**Table S3. Upregulated proteins in tears of pSS patients**

| **Upregulated proteins in pSS patients** | **Gene name** | **T-test**  **(p-value)** | **Spectral**  **count**  **control** | **Spectral**  **count**  **pSS** |
| --- | --- | --- | --- | --- |
| Ubiquitin-like modifier-activating enzyme 1 | UBA1 | 0.0001 | 229 | 312 |
| Fibrinogen beta chain | FGB | 0.0001 | 90 | 215 |
| Galectin-3 | LGALS3 | 0.0001 | 115 | 140 |
| Glutathione reductase, mitochondrial | GSR | 0.0001 | 47 | 95 |
| Elongation factor 1-gamma | EEF1G | 0.0001 | 23 | 51 |
| S-formylglutathione hydrolase | ESD | 0.0001 | 14 | 43 |
| Malate dehydrogenase, mitochondrial | MDH2 | 0.0001 | 12 | 40 |
| Copine-1 | CPNE1 | 0.0001 | 2 | 24 |
| Alcohol dehydrogenase [NADP(+)] | AKR1A1 | 0.00011 | 126 | 163 |
| Proteasome subunit alpha type-6 | PSMA6 | 0.00017 | 23 | 44 |
| Ig gamma-1 chain C region | IGHG1 | 0.00018 | 78 | 182 |
| Delta-aminolevulinic acid dehydratase | ALAD | 0.00023 | 18 | 31 |
| Ig gamma-3 chain C region | IGHG3 | 0.00023 | 49 | 105 |
| Rab GDP dissociation inhibitor beta | GDI2 | 0.00023 | 150 | 216 |
| Thioredoxin-dependent peroxide reductase, mitochondrial | PRDX3 | 0.00028 | 0 | 15 |
| Calpastatin | CAST | 0.00040 | 78 | 140 |
| Proteasome subunit beta type-9 | PSMB9 | 0.00042 | 6 | 23 |
| Echinoderm microtubule-associated protein-like 2 | EML2 | 0.00045 | 37 | 72 |
| Calreticulin | CALR | 0.00048 | 43 | 79 |
| Hsc70-interacting protein | ST13 | 0.00053 | 25 | 37 |
| Rab GDP dissociation inhibitor alpha | GDI1 | 0.00067 | 58 | 116 |
| Peptidyl-prolyl cis-trans isomerase B | PPIB | 0.00070 | 20 | 55 |
| Peroxiredoxin-1 | PRDX1 | 0.00071 | 130 | 177 |
| Protein disulfide-isomerase | P4HB | 0.00071 | 97 | 146 |
| Peroxiredoxin-2 | PRDX2 | 0.00074 | 63 | 76 |
| Protein SET | SET | 0.00078 | 36 | 61 |
| 5'(3')-deoxyribonucleotidase, cytosolic type | NT5C | 0.00099 | 15 | 41 |
| 3-mercaptopyruvate sulfurtransferase | MPST | 0.0010 | 12 | 32 |
| Moesin | MSN | 0.0010 | 14 | 42 |
| DNA-(apurinic or apyrimidinic site) lyase | APEX1 | 0.0011 | 1 | 23 |
| Glutaredoxin-1 | GLRX | 0.0012 | 15 | 31 |
| Puromycin-sensitive aminopeptidase | NPEPPS | 0.0013 | 129 | 222 |
| Fructose-1,6-bisphosphatase 1 | FBP1 | 0.0015 | 104 | 172 |
| 78 kDa glucose-regulated protein | HSPA5 | 0.0016 | 91 | 161 |
| Aldose 1-epimerase | GALM | 0.0016 | 30 | 50 |
| Mucin-5AC | MUC5AC | 0.0016 | 24 | 69 |
| Cadherin-1 | CDH1 | 0.0016 | 2 | 20 |
| Isocitrate dehydrogenase [NADP] cytoplasmic | IDH1 | 0.0017 | 117 | 184 |
| Glutathione synthetase | GSS | 0.0018 | 24 | 49 |
| Calcium-binding protein 39 | CAB39 | 0.0019 | 1 | 8 |
| Spectrin alpha chain, non-erythrocytic 1 | SPTAN1 | 0.0020 | 16 | 74 |
| Calpain-1 catalytic subunit | CAPN1 | 0.0021 | 149 | 212 |
| Vitamin D-binding protein | GC | 0.0023 | 62 | 115 |
| Transaldolase | TALDO1 | 0.0023 | 34 | 56 |
| Protein disulfide-isomerase A3 | PDIA3 | 0.0024 | 70 | 115 |
| Aldehyde dehydrogenase, dimeric NADP-preferring | ALDH3A1 | 0.0025 | 145 | 230 |
| Filamin-B | FLNB | 0.0025 | 13 | 63 |
| Septin-9 | SEPT9 | 0.0025 | 6 | 18 |
| Sciellin | SCEL | 0.0026 | 5 | 27 |
| Proteasome subunit beta type-8 | PSMB8 | 0.0029 | 10 | 36 |
| LIM domain only protein 7 | LMO7 | 0.0031 | 0 | 9 |
| Guanine nucleotide-binding protein G(i) subunit alpha-2 | GNAI2 | 0.0032 | 3 | 13 |
| Ubiquitin-40S ribosomal protein S27a | RPS27A | 0.0033 | 12 | 21 |
| Vinculin | VCL | 0.0035 | 69 | 122 |
| Proliferation-associated protein 2G4 | PA2G4 | 0.0036 | 12 | 27 |
| Aconitate hydratase, mitochondrial | ACO2 | 0.0038 | 0 | 19 |
| Alpha-actinin-4 | ACTN4 | 0.0040 | 375 | 532 |
| Cytosol aminopeptidase | LAP3 | 0.0042 | 110 | 156 |
| Ran-specific GTPase-activating protein | RANBP1 | 0.0042 | 3 | 14 |
| Cytosolic 10-formyltetrahydrofolate dehydrogenase | ALDH1L1 | 0.0044 | 29 | 65 |
| Protein AMBP | AMBP | 0.0047 | 19 | 37 |
| Plasma protease C1 inhibitor | SERPING1 | 0.0048 | 2 | 11 |
| Plasma kallikrein | KLKB1 | 0.0050 | 0 | 7 |
| Glyoxalase domain-containing protein 4 | GLOD4 | 0.0051 | 71 | 104 |
| Secernin-1 | SCRN1 | 0.0052 | 13 | 28 |
| Transketolase | TKT | 0.0053 | 60 | 97 |
| 14-3-3 protein sigma | SFN | 0.0053 | 143 | 209 |
| Citrate synthase, mitochondrial | CS | 0.0055 | 2 | 12 |
| Antithrombin-III | SERPINC1 | 0.0056 | 7 | 46 |
| Plastin-3 | PLS3 | 0.0057 | 106 | 142 |
| Fibrinogen alpha chain | FGA | 0.0062 | 72 | 128 |
| Cytosolic purine 5'-nucleotidase | NT5C2 | 0.0065 | 12 | 27 |
| Galactose-1-phosphate uridylyltransferase | GALT | 0.0065 | 0 | 6 |
| Adenosine kinase | ADK | 0.0065 | 25 | 52 |
| Proteasome subunit alpha type-3 | PSMA3 | 0.0069 | 18 | 30 |
| Myosin-9 | MYH9 | 0.0069 | 171 | 299 |
| Thioredoxin domain-containing protein 5 | TXNDC5 | 0.0070 | 0 | 7 |
| 14-3-3 protein epsilon | YWHAE | 0.0070 | 155 | 205 |
| Transitional endoplasmic reticulum ATPase | VCP | 0.0079 | 144 | 216 |
| Interferon-induced 35 kDa protein | IFI35 | 0.0081 | 1 | 8 |
| NSFL1 cofactor p47 | NSFL1C | 0.0082 | 32 | 45 |
| Protein disulfide-isomerase A4 | PDIA4 | 0.0082 | 11 | 51 |
| Neutrophil gelatinase-associated lipocalin | LCN2 | 0.0085 | 21 | 54 |
| Aspartate aminotransferase, cytoplasmic | GOT1 | 0.0085 | 3 | 20 |
| SH3 domain-binding glutamic acid-rich-like protein 2 | SH3BGRL2 | 0.0086 | 8 | 12 |
| Cytoplasmic dynein 1 intermediate chain 2 | DYNC1I2 | 0.0086 | 2 | 8 |
| Acyl-CoA-binding protein | DBI | 0.0088 | 54 | 86 |
| Complement C4-B | C4B | 0.0089 | 82 | 175 |
| Fructose-bisphosphate aldolase C | ALDOC | 0.0092 | 38 | 38 |
| Protein FAM49B | FAM49B | 0.0095 | 4 | 20 |
| Glucosamine-6-phosphate isomerase 1 | GNPDA1 | 0.010 | 15 | 32 |
| Ras-related protein Rab-11B | RAB11B | 0.010 | 11 | 25 |
| X-ray repair cross-complementing protein 6 | XRCC6 | 0.010 | 20 | 48 |
| Inositol monophosphatase 1 | IMPA1 | 0.011 | 21 | 40 |
| ATP synthase subunit beta, mitochondrial | ATP5B | 0.011 | 45 | 83 |
| 14-3-3 protein zeta/delta | YWHAZ | 0.011 | 155 | 212 |
| Osteoclast-stimulating factor 1 | OSTF1 | 0.011 | 4 | 16 |
| Glucose-6-phosphate isomerase | GPI | 0.012 | 76 | 142 |
| Periplakin | PPL | 0.012 | 46 | 93 |
| Heterogeneous nuclear ribonucleoprotein | HNRNPD | 0.012 | 17 | 27 |
| Complement C3 | C3 | 0.013 | 444 | 624 |
| Selenium-binding protein 1 | SELENBP1 | 0.013 | 145 | 206 |
| Dipeptidyl peptidase 3 | DPP3 | 0.013 | 90 | 155 |
| Barrier-to-autointegration factor | BANF1 | 0.013 | 9 | 19 |
| Mucin-1 | MUC1 | 0.013 | 5 | 13 |
| Quinone oxidoreductase | CRYZ | 0.014 | 24 | 46 |
| Annexin A3 | ANXA3 | 0.014 | 82 | 108 |
| Cystatin-A | CSTA | 0.014 | 0 | 7 |
| Hypoxanthine-guanine phosphoribosyltransferase | HPRT1 | 0.015 | 5 | 18 |
| Xaa-Pro dipeptidase | PEPD | 0.015 | 4 | 17 |
| Myosin light polypeptide 6 | MYL6 | 0.016 | 56 | 75 |
| Quinone oxidoreductase PIG3 | TP53I3 | 0.016 | 12 | 28 |
| L-lactate dehydrogenase B chain | LDHB | 0.016 | 26 | 49 |
| Annexin A2 | ANXA2 | 0.016 | 230 | 312 |
| Proteasome subunit alpha type-2 | PSMA2 | 0.016 | 26 | 41 |
| 14-3-3 protein gamma | YWHAG | 0.016 | 74 | 102 |
| Prolyl endopeptidase | PREP | 0.016 | 51 | 88 |
| Plastin-1 | PLSI | 0.016 | 48 | 70 |
| Nicotinate phosphoribosyltransferase | NAPRT | 0.017 | 59 | 93 |
| Proteasome subunit beta type-4 | PSMB4 | 0.017 | 22 | 49 |
| Alpha-aminoadipic semialdehyde dehydrogenase | ALDH7A1 | 0.017 | 9 | 25 |
| Protein disulfide-isomerase A6 | PDIA6 | 0.017 | 1 | 13 |
| Profilin-1 | PFN1 | 0.018 | 84 | 109 |
| Serine/threonine-protein phosphatase 2A activator | PPP2R4 | 0.018 | 20 | 28 |
| Stress-70 protein, mitochondrial | HSPA9 | 0.018 | 0 | 7 |
| Complement component 1 Q subcomponent-binding protein, mitochondrial | C1QBP | 0.018 | 2 | 12 |
| 14-3-3 protein eta | YWHAH | 0.018 | 58 | 97 |
| Serum albumin | ALB | 0.019 | 365 | 816 |
| Ig kappa chain C region | IGKC | 0.019 | 94 | 124 |
| Poly(ADP-ribose) glycohydrolase ARH3 | ADPRHL2 | 0.019 | 1 | 11 |
| Translin | TSN | 0.020 | 10 | 12 |
| Fumarate hydratase, mitochondrial | FH | 0.020 | 3 | 12 |
| Alpha-1-acid glycoprotein 1 | ORM1 | 0.020 | 28 | 66 |
| Eukaryotic initiation factor 4A-II | IF4A2 | 0.020 | 33 | 55 |
| Alpha-1-antitrypsin | SERPINA1 | 0.021 | 122 | 197 |
| Src substrate cortactin | CTTN | 0.021 | 30 | 42 |
| Fructose-bisphosphate aldolase A | ALDOA | 0.021 | 146 | 176 |
| Heterogeneous nuclear ribonucleoprotein H | HNRNPH1 | 0.021 | 4 | 14 |
| Endoplasmic reticulum aminopeptidase 1 | ERAP1 | 0.021 | 3 | 17 |
| Ig gamma-2 chain C region | IGHG2 | 0.022 | 40 | 74 |
| Ig mu chain C region | IGHM | 0.022 | 8 | 36 |
| Profilin-2 | PFN2 | 0.022 | 6 | 9 |
| Neuroblast differentiation-associated protein AHNAK | AHNAK | 0.022 | 82 | 150 |
| Glutathione S-transferase theta-1 | GSTT1 | 0.023 | 7 | 20 |
| Ubiquitin thioesterase OTUB1 | OTUB1 | 0.024 | 38 | 44 |
| Bifunctional purine biosynthesis protein PURH | ATIC | 0.024 | 83 | 130 |
| Triosephosphate isomerase | TPI1 | 0.025 | 138 | 147 |
| Complement factor H | CFH | 0.025 | 67 | 109 |
| DNA damage-binding protein 1 | DDB1 | 0.025 | 6 | 23 |
| 14-3-3 protein beta/alpha | YWHAB | 0.026 | 108 | 130 |
| Stress-induced-phosphoprotein 1 | STIP1 | 0.027 | 10 | 12 |
| Calmodulin-like protein 3 | CALML3 | 0.029 | 45 | 48 |
| Myeloid-derived growth factor | MYDGF | 0.025 | 3 | 11 |
| Geranylgeranyl transferase type-2 subunit alpha | PGTA | 0.025 | 1 | 7 |
| Latexin | LXN | 0.026 | 7 | 13 |
| Serine/threonine-protein phosphatase 2A catalytic subunit beta isoform | PPP2CB | 0.026 | 4 | 12 |
| Prelamin-A/C | LMNA | 0.027 | 32 | 68 |
| Hemopexin | HPX | 0.027 | 137 | 184 |
| 14-3-3 protein theta | YWHAQ | 0.028 | 80 | 121 |
| Alpha-1-antichymotrypsin | SERPINA3 | 0.028 | 20 | 35 |
| Thymidine phosphorylase | TYMP | 0.029 | 162 | 234 |
| Ezrin | EZR | 0.031 | 138 | 150 |
| Interleukin-1 receptor antagonist protein | IL1RN | 0.031 | 63 | 85 |
| 3´(2´),5´-bisphosphate nucleotidase 1 | BPNT1 | 0.031 | 0 | 4 |
| 6-phosphogluconolactonase | PGLS | 0.032 | 42 | 64 |
| UMP-CMP kinase | CMPK1 | 0.033 | 135 | 164 |
| Obg-like ATPase 1 | OLA1 | 0.033 | 12 | 23 |
| Solute carrier family 12 member 2 | SLC12A2 | 0.033 | 8 | 21 |
| 40S ribosomal protein S21 | RPS21 | 0.033 | 3 | 12 |
| Calnexin | CANX | 0.033 | 0 | 8 |
| PDZ and LIM domain protein 5 | PDLIM5 | 0.033 | 33 | 50 |
| Farnesyl pyrophosphate synthase | FDPS | 0.034 | 7 | 21 |
| Heat shock protein beta-1 | HSPB1 | 0.035 | 139 | 160 |
| Zinc finger protein 185 | ZNF185 | 0.035 | 14 | 26 |
| Keratin, type II cytoskeletal 5 | KRT5 | 0.036 | 97 | 166 |
| Rho GTPase-activating protein 1 | ARHGAP1 | 0.037 | 4 | 7 |
| Alpha-2-macroglobulin | A2M | 0.037 | 51 | 188 |
| Inorganic pyrophosphatase 2, mitochondrial | PPA2 | 0.038 | 0 | 5 |
| Complement C2 | C2 | 0.038 | 3 | 16 |
| Endoplasmic reticulum resident protein 29 | ERP29 | 0.039 | 3 | 17 |
| Macrophage migration inhibitory factor | MIF | 0.039 | 13 | 18 |
| Actin-related protein 3 | ACTR3 | 0.040 | 45 | 62 |
| ADP-ribose pyrophosphatase, mitochondrial | NUDT9 | 0.040 | 0 | 6 |
| Eukaryotic translation initiation factor 6 | EIF6 | 0.042 | 27 | 40 |
| Serine/threonine-protein kinase OSR1 | OXSR1 | 0.042 | 12 | 18 |
| 26S proteasome non-ATPase regulatory subunit 13 | PSMD13 | 0.042 | 17 | 25 |
| Heat shock 70 kDa protein 1A | HSPA1A | 0.044 | 295 | 323 |
| F-actin-capping protein subunit alpha-2 | CAPZA2 | 0.044 | 51 | 67 |
| 26S proteasome non-ATPase regulatory subunit 11 | PSMD11 | 0.044 | 4 | 10 |
| Thioredoxin domain-containing protein 17 | TXNDC17 | 0.045 | 25 | 39 |
| Cytosolic non-specific dipeptidase | CNDP2 | 0.045 | 179 | 192 |
| Eukaryotic translation initiation factor 2 subunit 2 | EIF2S2 | 0.045 | 3 | 13 |
| Kynureninase | KYNU | 0.047 | 47 | 91 |
| 14 kDa phosphohistidine phosphatase | PHPT1 | 0.047 | 30 | 47 |
| Hepatoma-derived growth factor | HDGF | 0.048 | 13 | 21 |
| Elongation factor 1-beta | EEF1B2 | 0.048 | 6 | 15 |
| Prefoldin subunit 2 | PFDN2 | 0.048 | 1 | 7 |
| Complement component C8 gamma chain | C8G | 0.048 | 1 | 8 |
| Phosphoglucomutase-2 | PGM2 | 0.049 | 8 | 14 |
| Ras GTPase-activating-like protein IQGAP1 | IQGAP1 | 0.049 | 208 | 263 |
| Proteasome subunit alpha type-1 | PSMA1 | 0.050 | 22 | 27 |
