# Supplementary figures and images for: Identification of potential saliva and tear biomarkers in primary Sjögren’s syndrome, utilising the extraction of extracellular vesicles and proteomics analysis
Source: Arthritis Res Ther. 2017 Jan 25;19:14. doi: 10.1186/s13075-017-1228-x (PMC5264463; doi:10.1186/s13075-017-1228-x)

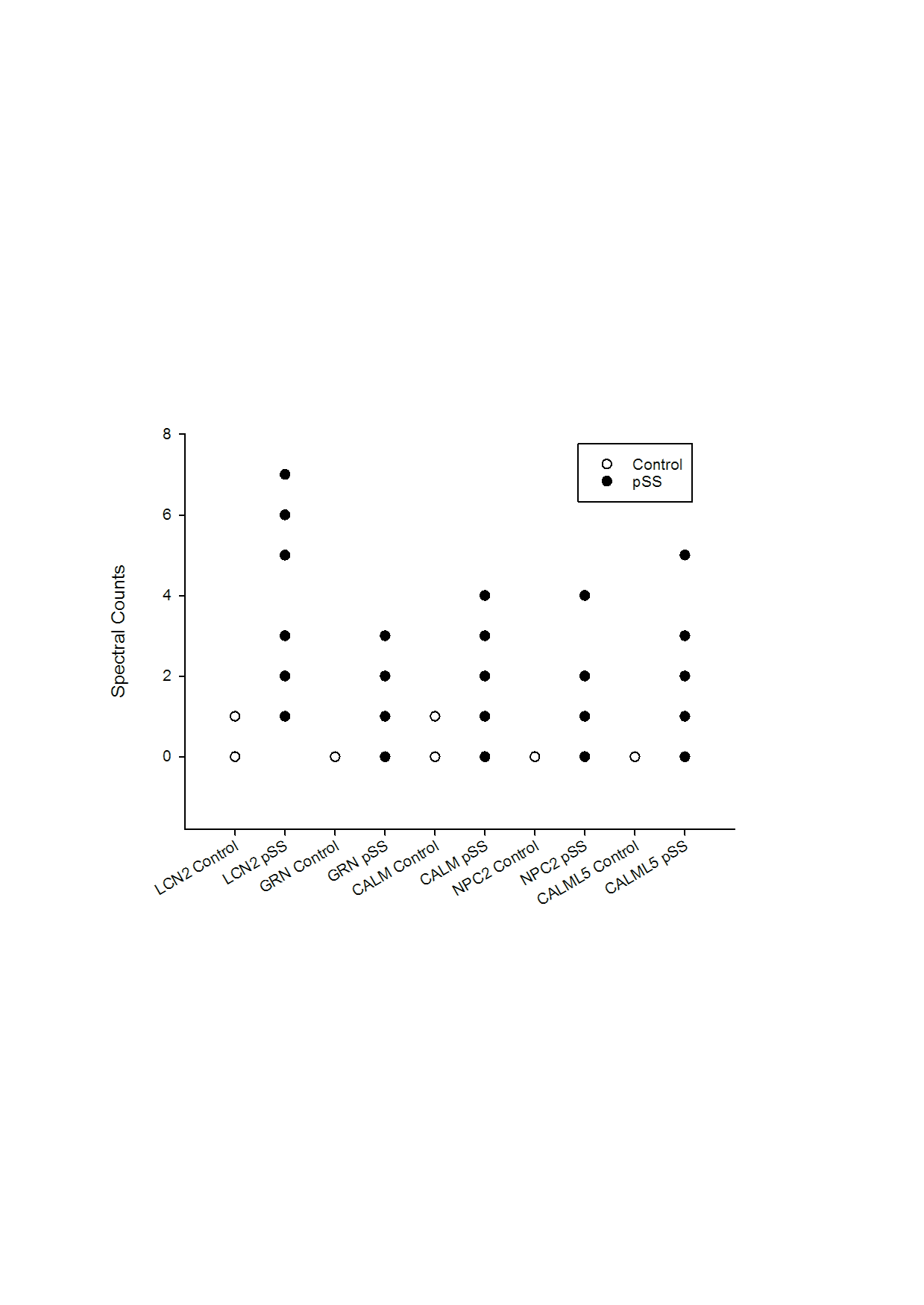

Supplement: Additional file 2: Figure S1. — Spectral count representation of highly upregulated pSS-associated proteins identified in stimulated whole saliva. Spectral counts from individual stimulated whole saliva samples from patients with pSS (black circles) and controls (white circles) showing little or none of the proteins LCN2, GRN, CALM, NPC2 and CALML5 in controls compared to patients with pSS (TIF 8483 kb) [file 13075_2017_1228_MOESM2_ESM.tif]

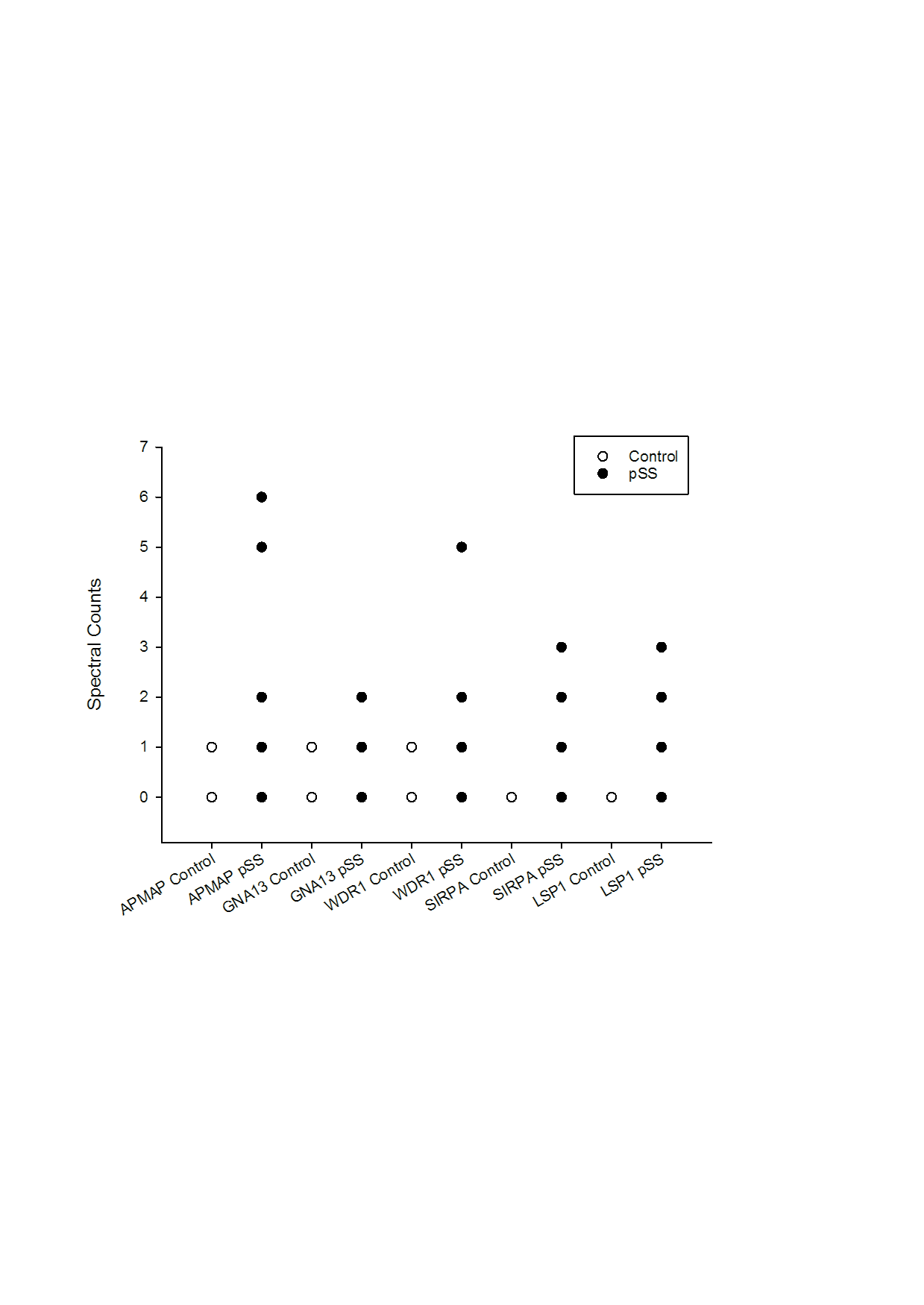

Supplement: Additional file 3: Figure S2. — Spectral count representation of highly upregulated pSS-associated proteins detected in EVs from whole saliva. Spectral counts from individual EV samples isolated from whole saliva from patients with pSS (black circles) and controls (white circles) showing little or none of the proteins APMAP, GNA13, WDR1, SIRPA and LSP1 in controls compared to patients with pSS (TIF 8483 kb) [file 13075_2017_1228_MOESM3_ESM.tif]

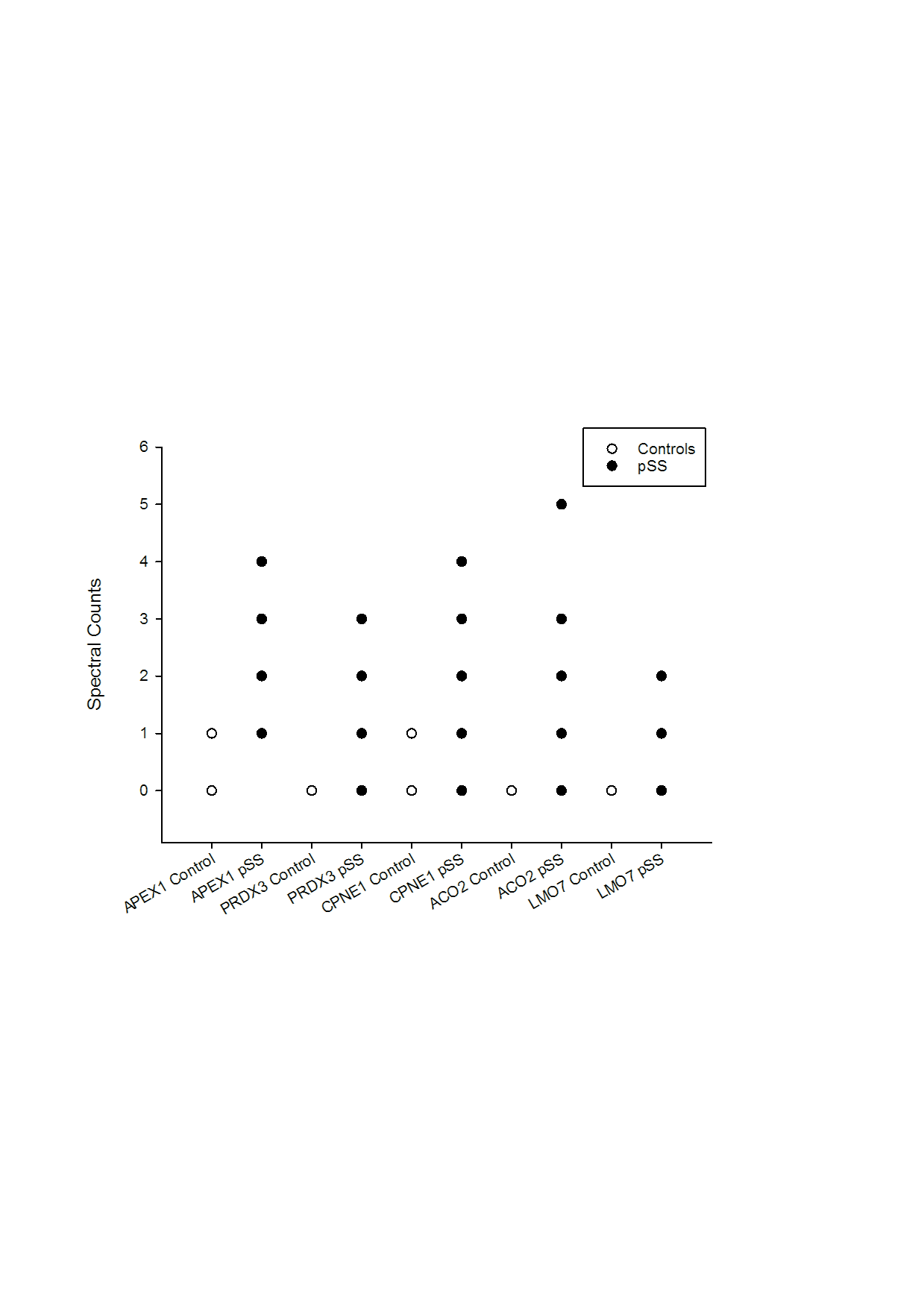

Supplement: Additional file 4: Figure S3. — Spectral count representation of highly upregulated pSS-associated proteins identified in tear fluid. Spectral counts from individual tear samples of patients with pSS (black circles) and controls (white circles) showing little or none of the proteins APEX1, PRDX3, CPNE1, ACO2 and LMO7 in controls compared to patients with pSS (TIF 8483 kb) [file 13075_2017_1228_MOESM4_ESM.tif]
